# Elucidation of EEG Characteristics of Fuzzy Reasoning-Based Heuristic BCI and Its Application to Patient With Brain Infarction

**DOI:** 10.3389/fnbot.2020.607706

**Published:** 2021-01-25

**Authors:** Norihiko Saga, Atsushi Doi, Teruo Oda, Suguru N. Kudoh

**Affiliations:** Department of Human System Interaction, School of Science and Technology, Kwansei Gakuin University, Sanda, Japan

**Keywords:** ankle rehabilitation, brain-computer interface (BCI), EEG, fuzzy template matching, neurorehabilitation

## Abstract

Non-invasive brain–computer interfaces (BCIs) based on common electroencephalography (EEG) are limited to specific instrumentation sites and frequency bands. These BCI induce certain targeted electroencephalographic features of cognitive tasks, identify them, and determine BCI's performance, and use machine-learning to extract these electroencephalographic features, which makes them enormously time-consuming. In addition, there is a problem in which the neurorehabilitation using BCI cannot receive ambulatory and immediate rehabilitation training. Therefore, we proposed an exploratory BCI that did not limit the targeted electroencephalographic features. This system did not determine the electroencephalographic features in advance, determined the frequency bands and measurement sites appropriate for detecting electroencephalographic features based on their target movements, measured the electroencephalogram, created each rule (template) with only large “High” or small “Low” electroencephalograms for arbitrarily determined thresholds (classification of cognitive tasks in the imaginary state of moving the feet by the size of the area constituted by the power spectrum of the EEG in each frequency band), and successfully detected the movement intention by detecting the electroencephalogram consistent with the rules during motor tasks using a fuzzy inference-based template matching method (FTM). However, the electroencephalographic features acquired by this BCI are not known, and their usefulness for patients with actual cerebral infarction is not known. Therefore, this study clarifies the electroencephalographic features captured by the heuristic BCI, as well as clarifies the effectiveness and challenges of this system by its application to patients with cerebral infarction.

## Introduction

Brain–computer interfaces (BCIs), which are interfaces for detecting cerebral activity and controlling devices, are mainly used for EEG (electroencephalogram). In recent years, BCI has been used as a technique for manipulating rehabilitation equipment for stroke patients and human support equipment in the welfare engineering field (Wolpaw R. J. et al., 2000; Ahn et al., 2013; Foong et al., 2019). In the research applications of BCI using these EEGs, it is mainstream to detect known EEG features by machine learning, which are expressed in specific cognitive activities, and to treat this as a control signal of equipment.

Examples of applications of the BCIs include, for example, those that provide visual stimuli that call up event-related potential brain activity (ERPs) (Liu et al., 2000; Mattout et al., 2015) or stable-state visual evoked potentials (SSVEP) (Andrew et al., 2013; Norcia et al., 2015), but are difficult to include motion intentions or motor images that have such factors as the orientation and velocity of body movement.

Another example is BCIs that use frequency information (event-related synchronization/desynchronization, ERS/ERD) (Pfurtscheller et al., 2000; Wolpaw J. et al., 2000; Kudoh et al., 2008; Joen et al., 2011) that uses EEG synchronous and asynchronous components associated with an event. This is characterized by an electroencephalogram in which the power in the specific frequency band of the α wave and β wave rises/decreases in the motion image (Pfurtscheller and Da Silva, 1999). However, although there are individual differences, ERD/ERS tend to be difficult to develop if there is no prior training, and many have been experimented with after several months or more of training (Wolpawand and Wolpaw, 2012; Shanechi et al., 2016).

Even for the EEG characteristics at the time of motion image recall, which are considered to be relatively reproducible, it is common that the accuracy of EEG feature detection by BCI is greatly affected by the way images are recalled and the success or failure of training (Ruby and Decety, 2001; Xu et al., 2019).

Therefore, we developed a BCI system (Oda and Kudoh, 2017, 2018) that extracts the motion intention more easily and quickly by simply patterning the increase/decrease of the amplitude of the EEG power using a template matching method (FTM) based on fuzzy reasoning, without using a technique to detect known EEG feature patterns in specific measurement sites and frequency bands.

This paper clarifies the electroencephalographic features captured by the developed heuristic BCI, as well as clarifies the effectiveness and challenges of this system from its application situation to patients with cerebral infarction.

## Heuristic BCI Systems

Heuristic BCI used in this study differs greatly from the image-recognition FTM (fuzzy template matching) (Fukuda et al., 1995; Yachida et al., 1999; Li et al., 2001)in that it has a process of creating templates without using the peak values of the membership function and pruning process. Details have been described in the developed ankle neural rehabilitation system (Saga et al., 2019), which is outlined here.

**Figure 1:**
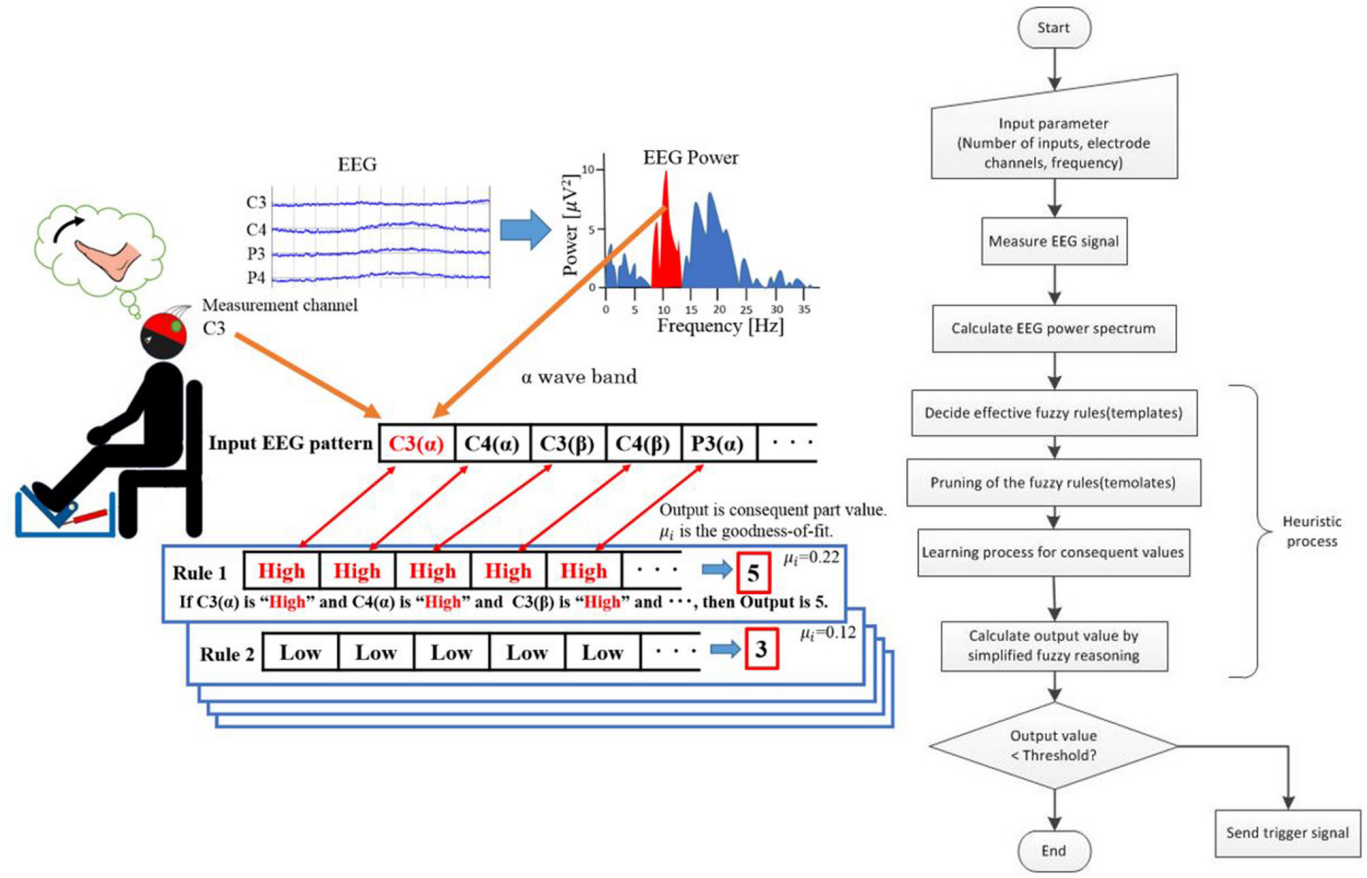
Overview of heuristic BCI (template matching method based on fuzzy reasoning).

A simplified reasoning method (Takagi and Sugeno, 1985) is used as a framework of fuzzy reasoning, and each template and output value are correlated by learning of the consequent part values. Since inputs may be anything as long as they can be expressed as values or degrees are high or low, templates can be constructed for various inputs with different features. In this study, two different cognitive tasks are targeted: a resting state and an imaginary state with moving foot. For example, it is possible to flexibly configure a search space for a combination of inputs having greatly different characteristics and reference values, such as “electroencephalogram power value of α-wave frequency band of measurement part C3,” “electromyography of right arm,” “size of body movement,” and the like. In addition, since the fuzzy number is used, it is not necessary to set or normalize a clear threshold value for the input. The antecedent part of each rule can construct a pattern in which fuzzy labels of either High or Low are used as elements for each entry, and any combination can be prepared. For example, for eight inputs, a maximum of 2^8^ = 256 rules can be constructed for all combinations of element patterns. Since simplified fuzzy reasoning (Takagi and Sugeno, 1985) is used, the consequent part shall be singleton and real value. The value to be output to BCI when a certain cognitive condition is activated is used as an teacher signal, and the consequent part value of each rule is determined by learning. This operation links the brain activity with the value to be output. In this study, we first took initial measurements of each input as data for learning and calculated the compatibility degree of each rule (template) to these data. The output value is calculated by the weighted average of the consequent part values weighted by the compatibility degree of each rule (template). This method is similar to learning-type simplified fuzzy reasoning. In heuristic BCI of this study, a rule which is valid for state discrimination is automatically selected by learning the real value of the consequent part with the output value as a teacher signal. This learning is a process in which a search space composed of multiple rules (templates) defined by measurement parts and fuzzy labels is exhaustively searched, and effective rules are discovered Figure 1 shows the overview of heuristic BCI (template matching method based on fuzzy reasoning). The left side of Figure 1 shows the outline from EEG measurement to preparation of the fuzzy template, and the right side of Figure 1 shows the flow diagram of the entire heuristic BCI. The algorithms for calculating the actual Heuristic BCI are shown below.

First, fuzzy-label placement patterns are used in the anterior part of fuzzy rules (templates), which combine two-value fuzzy labels, High and Low, that ambiguously express their intensities, for electroencephalographic power inputs defined by combinations of electroencephalographic measurement channels and frequency bands. An initial value of this template constitutes a rule with all combinatorial patterns in the front part composed of two labels. Electroencephalogram data obtained by the initial measurement of EEG are fast Fourier transformed (FFT), and the area of the amplitude power spectrum is summed with the frequency bands in the preset ranges to serve as input values.

The membership function exists only in the number of labels for each input sequence element and is set based on the maximum and minimum values of the input range of the membership function. The membership value is calculated for each label by this membership function, the compatibility degree of each rule for the entered pattern is calculated, and the output value is the weighted average of the posterior part value of each rule with this compatibility degree as the weight.

As shown in Equation (1), the membership value is calculated by inputting the electroencephalographic power value into the membership function corresponding to the fuzzy label for each entry of the *i* th rule, and multiplying the membership value *G*_*j*_ at each input value *j* is the compatibility degree μ_*i*_.

(1) $$\mu_{i}=\prod_{j=0}^{n}G_{j}$$

The output value *Z* is calculated considering compatibility degree as in Equation (2). Here *Z*_*i*_ is the posterior case value of the *i* th rule.

(2) $$Z=\frac{\sum_{i=1}^{n}\left(\mu_{i}*Z_{i}\right)}{\sum_{i=1}^{n}\mu_{i}}$$

The output values correspond to the relative compatibility degree during motor imagery, and those with higher output values are automatically generated as patterns during motor imagery, but some of the generated rules include rules that resulted in high compatibility degree during both motor imagery and rest. The rules that became highly fit in both are responsible for the decreased accuracy of discrimination, and pruning is performed to remove this rule. Pruning is the processing which removes the rule that the compatibility degree must be high in both motor imagery and rest and retains the rule that compatibility degree must be high for either. The sum of the compatibility degree during motor imagery and the resting state for each rule is obtained and the maximum *Ob*_(*i*)_ is determined by Equation (3).

(3) $$Ob_{\left(i\right)}=max\left\{\sum_{t=1}^{te}Ot_{\left(i,t\right)},\sum_{t=1}^{te}On_{\left(i,t\right)}\right\}$$

Here, *t* is the input time, *te* is the maximum value of the input time, and *Ot*_(*i,t*)_, *On*_(*i,t*)_ are the ith compatibility degree in motor imagery and time t at rest, respectively. Then, the difference in the sum of each compatibility degree is calculated as shown in Equation (4), and the absolute *Os*_(*i*)_ is calculated.

(4) $$Os_{\left(i\right)}=\left|\sum Ot_{\left(i\right)}-\sum On_{\left(i\right)}\right|$$

If the value normalized by *Ob*_(*i*)_ is smaller than the threshold set by *Ob*_(*i*)_, the *i* th rule based on Equation 5 is removed with high compatibility degree in both motor imagery and the resting state. If *Ob*_(*i*)_ is greater than the threshold, it is considered to have a high compatibility degree only for each factor, and the rule is retained.

(5) $$Pruning\left(R_{\left(i\right)}\right)=\{\begin{array}{c} retain,if\left(\frac{Os_{\left(i\right)}}{Ob_{\left(i\right)}}\geq th\right)\\ delete,if\left(\frac{Os_{\left(i\right)}}{Ob_{\left(i\right)}}<th\right) \end{array}$$

Only a subset of the retained templates is used to perform the learning process with the input values stored for pruning. The trailing values of a subset of rules are set by the learning process and computed using the new rule set. The learning process for posterior values uses the steepest descent method and is updated as shown in Equation (6). Here, ${Z^{\prime }}_{i}$ is the posterior part value before updating, T is the teacher signal (target value), ρ is the learning coefficient, and the learning coefficient is set at 0.9.

(6) $$Z_{i}={Z^{\prime }}_{i}+\rho^{*}\mu_{i}*\left(T-Z\right)$$

The teacher signal enters the electroencephalogram pattern which appears in the motor imagery in FTM, the value which is to be labeled in this electroencephalogram becomes the teacher signal, and the arbitrary numerical value can be set.

In the above procedure, an effective rule is created to identify motor imagery.

When the output value obtained by electroencephalography measurement after the rule selection exceeds the set threshold, the trigger signal rises, and it is set so that the signal is sent to the operation program of the rehabilitation equipment.

## Ankle Neurorehabilitation System

We developed a neurorehabilitation system for rehabilitation training of pneumatic cylinder driving to detect electroencephalographic patterns of movement imagery from electroencephalography (EEG) using Heuristic BCI and performed dorsiflexion movements of the ankle joint and performed motion assessment of the system. This system measures motor imagery which dorsiflexes the right foot in the initial measurement of the electroencephalogram and rests in the relaxed state. After the initial measurement was completed, FTM and learning were performed and learning. The learning was completed in 1 ~ 2 min, the electroencephalography measurement was carried out again, and the motion signal was sent to the rehabilitation equipment when the motor imagery was detected, and it becomes a system in which the rehabilitation equipment (Saga and Saito, 2008; Saga et al., 2011) operates.

## Materials and Methods

### Participants

The total volunteers were 10 healthy subjects in their 20s and 1 case patient with stroke hemiplegia who were seated in chairs with as much relaxation as possible. The subjects placed their right foot in the foot position of an ankle rehabilitation device, and were instructed not to move, except for at the designated joints. An LED (light emitting diode) for timing instructions was placed near the toes. The stroke hemiplegic patient were 6 months from the onset of symptoms, and the degree of paralysis of the lower limbs was Brunnstrom stage III, in which collaborative movements are strongly manifested.

The studies involving human participants were reviewed and approved by Kwansei Gakuin University Institutional Review Board for the Protection of Human Subjects of Medical Research (KGIRB). The patients/participants provided their written informed consent to participate in this study.

### Experimental Conditions

Electroencephalography was measured using a multi-channel digital electroencephalography measurement system (made by Active Two System, Biosemi) for healthy subjects. For the case patient, 6 electrodes were placed in total at C3, Cz, C4, P4, Pz, and P3 in the extended 10–20 method, which is active during movement-related activities. The frequency bands to be measured were α-wave (8–12 Hz) and β-wave (13–30 Hz), which are characterized by potential decrease in ERD. A reference electrode was placed near the top of the head and used unipolar dielectric method of measurement, and the sampling frequency was set at 2,048 Hz. Electrodes used 5 mm diameter silver–silver chloride active electrodes, and conductive pastes were applied to the contact surfaces.

In a rehabilitation system in which the ankle joint rehabilitation equipment moves, the electroencephalogram features which show the motor imagery are detected by Heuristic BCI using FTM.

During the electromyography, a disposable electrode (LecTrode NP, Advance) was attached to the surface of the tibialis anterior muscle, which exerts muscle strength during dorsiflexion of the right ankle joint, with the reference electrode placed on the right lateral malleolus after swabbing the skin with alcohol at the placement site of the electrode. An active electrode was connected to the electrode, and data was sent to a personal computer by a transmitter (MARQ, manufactured by Kisseikomtec) and recorded. A Vital Recorder (manufactured by Kisseikomtec) was used for recording, and the sampling frequency was set at 2,048 Hz.

### Experimental Procedures

This experiment was developed to discriminate between motor imagery with dorsiflexion of the right foot and resting state.

As a teaching method for the motor imagery, the patient was asked to dorsiflex the right foot for 5–10 min, and the practice was carried out until imagery of the dorsiflexion was possible. This practice was set as Task 0. Since it was reported that it is difficult to imagery dorsiflexion and dorsiflexion of the right foot, a paralyzed limb, in the case patient, the system was trained until it was possible to image dorsiflexion of the paralyzed limb while viewing the dorsiflexion of the left foot, a healthy limb. After confirming that motor imagery was possible, motor imagery and resting state were measured for 2 min each as learning tasks in FTM. This was Task 1.

After completion of the measurement of the learning task, the resting state and motor imagery were repeated twice, alternating for 2 min as Task 2. At this time, when motor imagery was identified, the ankle rehabilitation device was set to operate in the dorsiflexion direction for 15 s. Motor imagery was performed by flashing the LED with a 5-s cycle. In healthy subjects, the motion angle was set to dorsiflex 30 degrees from 45 degrees of plantar flexion to 15 degrees of plantar flexion, while in case patient it was set to dorsiflex 15 degrees from 45 degrees of plantar flexion to 30 degrees of plantar flexion, taking safety into account.

### Electroencephalography/Myopotential Processing and Analysis Methods

As a pre-processing, the EEG signal was notch filtered in order to exclude 60 Hz of electrical noise, which was in turn band-pass filtered with the frequencies between 0.04 and 200 Hz (FIR filtering). The frequency was chosen to include the full spectrum of the EEG signal, considering the correlation between EEG and EMG (Tanaka and Saga, 2018; Tanaka et al., 2018) and excluding high frequencies from the EMG. Then, we conducted the independent component analysis (ICA) to whole EEG signals in order to segregate motor related signals from the artifact components of Electrooculography (EOG) such as eye blink. We used EEGLAB (Delorme and Makeig, 2004) operated on Matlab (Mathworks, Boston, USA). In addition, frequency analysis was performed to extract time features at each measurement location. The myopotential was processed by root mean square (Root Mean Square, RMS), and then the moving averages were performed. We also performed normalization to processed data with the aim of eliminating individual differences in electroencephalography and myogenic potentials.

## Results and Discussion

### Healthy Subjects

**Figure 2:**
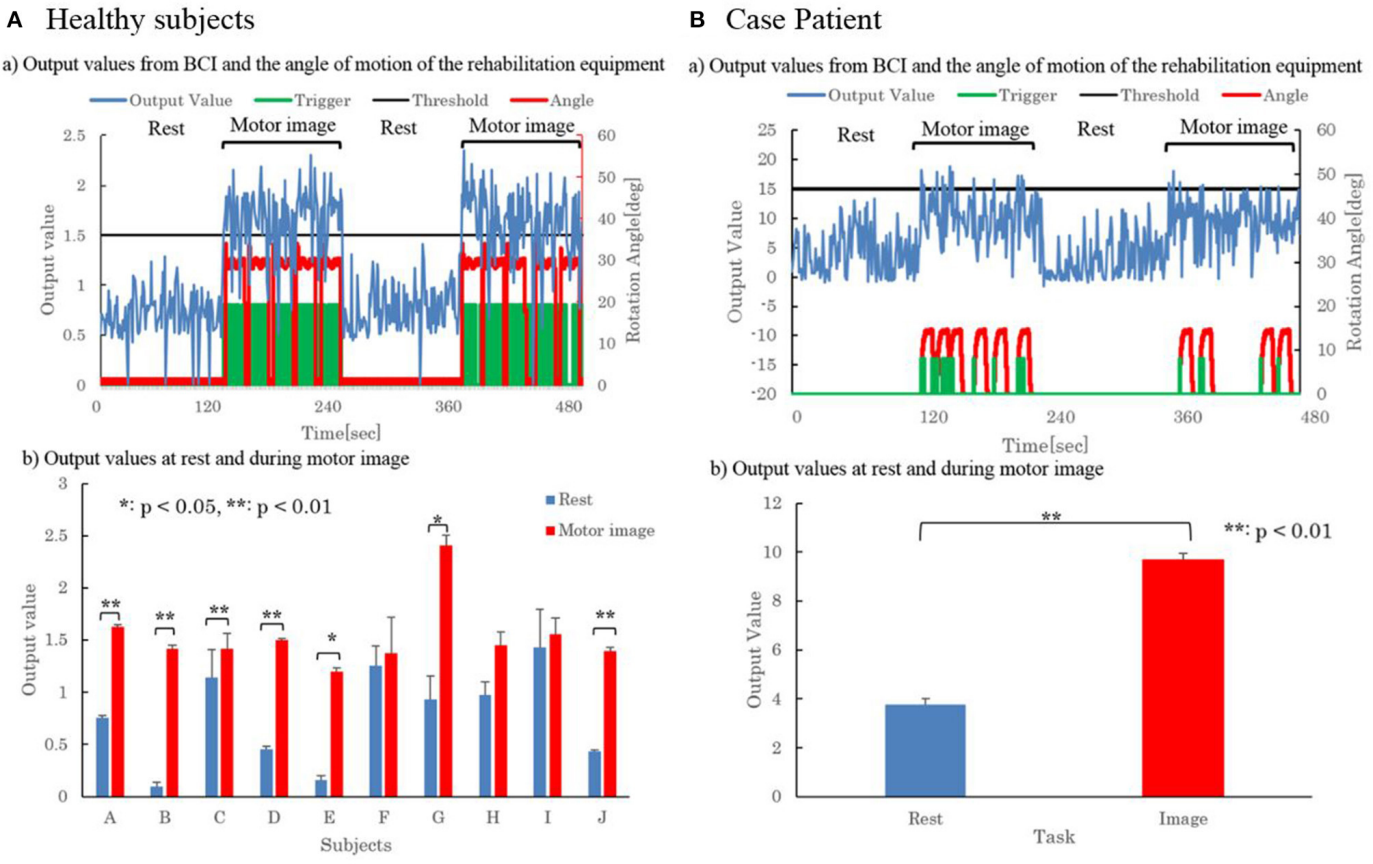
Motor Image Detection from BCI and the operation of the rehabilitation system. **(A)** Shows the results of 10 healthy subjects and **(B)** shows the results of one case patient.

Figure 2A(a) shows the output values obtained with the FTM of one representative subject in Task 2. From the figure, it can be confirmed that at rest, the output value was below the threshold, and in motor imagery, the output value was above the threshold.

Since the motion angle of the rehabilitation equipment rose when the trigger signal was generated, it was confirmed that the rehabilitation equipment was operating when the motor imagery is detected.

Shown in Figure 2A(b) are the summed averaged output values during rest and motor imagery for each of the 10 subjects in Task 2. Error bars in the figure indicate the standard deviation. A *t*-test was performed at rest and during motor imagery, respectively, resulting in two subjects with a *p*-value of 0.05 or less and five subjects with a *p*-value of 0.01 or less. Seven of the 10 subjects showed significant differences at rest and during motor imagery, confirming the detection of motor imagery by EEG.

Table 1 shows the results of extracting the top five rules with higher posterior values after performing FTM for one healthy subject and ordering them in order of increasing posterior values.

**Table 1:** Top five rules with higher consequent part values in healthy subjects.

**C3 (α wave)**	**C3 (β wave)**	**Cz (α wave)**	**Cz (β wave)**	**C4 (α wave)**	**C4 (β wave)**	**P3 (α wave)**	**P3 (β wave)**	**P_**Z**_ (α wave)**	**P_**Z**_ (β wave)**	**P4 (α wave)**	**P4 (β wave)**
High	High	High	High	High	High	High	Low	High	High	High	High
High	High	Low	High	High	Low	High	High	High	High	High	High
Low	High	Low	Low	High	Low	High	Low	High	Low	Low	High
Low	High	Low	High	Low	Low	Low	Low	Low	Low	Low	Low
Low	High	Low	Low	High	Low	Low	High	High	Low	Low	High

In 10 subjects, the top five consequent part values were extracted in the same manner and included the number of “High” fuzzy labels with high electroencephalogram powers. The results were extracted as combinations of measurement points and frequency bands in which the β wave of C3, the α wave of C4, and the α wave of Pz contributed to discrimination.

For the three extracted electroencephalograms and myopotentials in Task 2, the time issued by LED, a cue for action initiation, for 10-min movements during motor imagery was taken in the time range from −5 to 20 s as 0 s, and the summed averaged results are shown in Figure 2A(a). The dashed line in the diagram shows the mean from −4 to −1 s; this dashed line is used as a threshold to discriminate between decreases or increases in EEG power. From the results, it was confirmed that the potential of the β wave of C3 was lowered in the event of around 0 s. The peak in the positive direction was confirmed around 1 ~ 2 s, and it lowered to about 13 s. In the α wave of P3, the lowering of the potential was confirmed from around −1 s.

The potential rose prior to the rise of the myopotential, and the peak position resulted mostly in agreement with the peak position of the myopotential. In the αwave of Pz, an increase in the potential was identified for events around 0 s. After the potential lowered in about 1 s, the potential rose in about 2 s preceding the muscle potential, it lowered in about 5 s once, and it became the result which corresponded with the amplitude of the muscle potential in 7 ~ 13 s.

**Figure 3:**
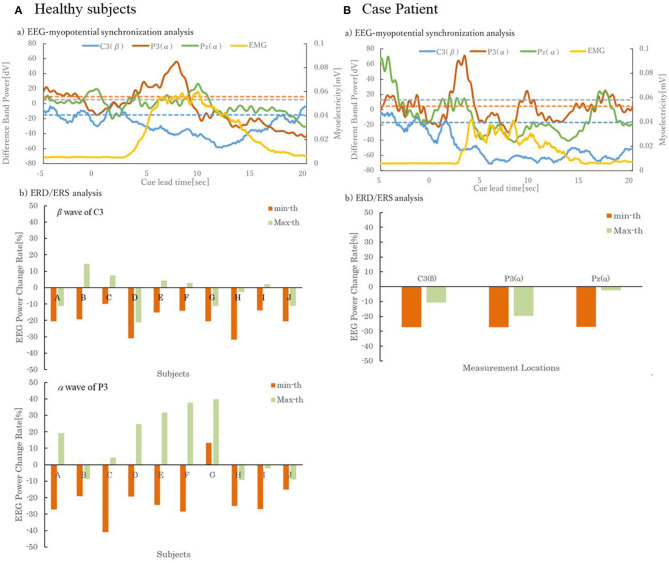
Analysis of EEG and EMG. **(A)** Shows the results of 10 healthy subjects and **(B)** shows the results of one case patient.

Regarding the alpha waves of C3 and P3, where the electroencephalographic power had decreased in the event to around 0 s [Figure 3A(a)], ERD/ERS was considered to be emerging, and the minimum and maximum values of the percentage change in electroencephalographic power at 0–1 s were determined. The results for 10 subjects are shown in Figure 3A(b), where the ERD was defined as the negative value when the difference between the minimum and the threshold was taken from the minimum value (min-th) and the ERS was defined as the positive value when the difference between the maximum and the threshold was taken from the maximum value (max-th). From these results, ERD was identified in all subjects at the C3 beta wave and in subjects other than Subject G at the P3 alpha wave. Five persons in β wave of C3 and 6 persons in α wave of P3 were observed by ERS.

### Case Patient

Figure 2B(a) shows the output values obtained with FTM in Task 2. It can be confirmed from the figure that the output value was below the threshold in the case patient as well as the healthy subjects at rest, and the output value was above the threshold in the motor imagery. Further, from the shift of the operation angle of the trigger signal and the rehabilitation device, a trigger signal was output when detecting the motion image, and it could be confirmed that the rehabilitation device was operating accordingly.

Table 2 shows the results of extracting the top five of the rules with higher posterior values after performing FTM for one subject and ordering them in order of increasing posterior values. When counting the number of fuzzy-label high, which had increased electroencephalographic power, α and β waves of C3 and CZ, β waves of C4, α and β waves of P3, and α waves of Pz were extracted as combinations of measurement sites and frequency bands contributing to discrimination.

**Table 2:** Top five rules with higher consequent part values in case patient.

**C3 (α wave)**	**C3 (β wave)**	**Cz (α wave)**	**Cz (β wave)**	**C4 (α wave)**	**C4 (β wave)**	**P3 (α wave)**	**P3 (β wave)**	**P_**Z**_ (α wave)**	**P_**Z**_ (β wave)**	**P4 (α wave)**	**P4 (β wave)**
High	High	High	High	High	High	High	High	High	High	High	High
Low	High	High	Low	High	High	High	High	High	High	Low	Low
High	Low	High	High	Low	Low	Low	Low	High	Low	Low	Low
Low	Low	Low	Low	Low	Low	Low	Low	Low	Low	Low	Low
High	High	Low	High	Low	High	High	High	High	Low	Low	Low

The summed averaged results for rest and motor imagery, respectively, at the output value in Task 2 are shown in Figure 2B(b). Error bars in the figure indicate the standard deviation. From this result, it was confirmed that the significance was obtained, since the *p*-value was <0.01, when the *t*-test was carried out in the resting state and with motor imagery.

Figure 3B(a) shows the electroencephalography and muscle potentials in Task 2. The time issued by LED, a cue for action initiation, for 10-min movements during motor imagery was measured the time range from −5 to 20 s as 0 s, and the summed averaged results are shown. Since there was one case patient, the β wave of C3, α wave of P3, and α wave of Pz are displayed as in healthy subjects. In the β wave of C3, the lowering of the potential was confirmed from around −1 s. A peak in the positive direction was identified around 2 s, and then decreased. In the α wave of P3, a decrease in the potential was identified from around −1 s. It rose in the form which precedes the muscle potential in about 1 s, and it became the amplitude which was similar to the muscle potential in about 8 s. It was confirmed that the potential lowered from around −4 s in the α wave of Pz, and it rose from around 0 s. Peaks preceded the myogenic potential by about 3 s, but at about 4–8 s, the amplitude of the myogenic potential was similar to that of the myogenic potential.

As shown in Figure 3B(b) and (c), in which ERD/ERS analysis was performed on 10 healthy subjects, the minimum and maximum changes in EEG power between 0 and 1 s were determined, and the ERD was defined as negative when the difference between the minimum and the threshold was obtained (min-th), and the ERS was defined as positive when the difference between the maximum and the threshold was obtained (max-th). The results of ERD/ERS analysis are shown in Figure 3B(b). Though ERD was confirmed in all measurement positions from the result, ERS was not confirmed.

## Conclusion

In the neurorehabilitation system using the proposed Heuristic BCI with fuzzy reasoning, it was confirmed that motor imagery dorsiflexing the right foot and resting-state can be distinguished with high accuracy not only in healthy subjects but also in the case patient, and that it is a rehabilitation system that can be used in motor imagery training. The stroke hemiplegic patient was able to experience the first time of symptoms that foot dorsiflexion actually occurs with the intention since the onset.

From the experimental results of the healthy subjects and case patient, it was confirmed that C3, the primary motor area, and P3, is the somatosensory area, contributed to the discrimination when the combination of measurement position and frequency band labeled as High was extracted from the top of the rule where the posterior part value increased with the combination of measurement position and frequency band, which seemed to contribute to the discrimination. P3 and Pz, which are both part of the somatosensory area, contributed to the discrimination, and the ERD was confirmed in the β wave of C3 and α wave of P3 from the synchronization analysis result of the electroencephalogram and muscle potential. This suggests that this system based on fuzzy inference discriminates ERD as a detection object in a short period of time by BCI using machine learning, which is commonly used in previous studies, and it is a system that can be distinguished by a few minutes of imagery training and can be expected to maintain and increase muscle strength.

In this study, the target was made to be the position of the first motor area and somatosensory area, α wave and β wave, and the application case of this system of the case patient will be increased in future, and the effectiveness of the system will be confirmed by examining whether the effective channel exists in other position and frequency band with the aim of further accuracy improvement and time shortening, and by advancing the narrowing of measurement channel number and frequency.

## Data Availability Statement

The raw data supporting the conclusions of this article will be made available by the authors, without undue reservation.

## Ethics Statement

The studies involving human participants were reviewed and approved by Kwansei Gakuin University Institutional Review Board for the Protection of Human Subjects of Medical Research (KGIRB). The patients/participants provided their written informed consent to participate in this study.

## Author Contributions

NS designed this rehabilitation system and wrote the manuscript. AD performed the experiments. SK designed this BCI algorithm and supervised the work. All authors contributed to the article and approved the submitted version.

## Conflict of Interest

The authors declare that the research was conducted in the absence of any commercial or financial relationships that could be construed as a potential conflict of interest.
